# Physiological and Biochemical Effects of Echium Amoenum Extract on Mn^2+^-Imposed Parkinson Like Disorder in Rats 

**DOI:** 10.15171/apb.2018.079

**Published:** 2018-11-29

**Authors:** Leila Sadeghi, Farzeen Tanwir, Vahid Yousefi Babadi

**Affiliations:** ^1^Department of Animal Biology, Faculty of Natural Sciences, University of Tabriz, Tabriz, Iran.; ^2^Matrix Dynamics Group, University of Toronto, Canada.; ^3^Department of Physiology, Payam Noor University of Iran, Iran.

**Keywords:** Catecholamine, Cognitive disorder, Depression like behavior, Hippocampus, Manganism, Mitochondria dysfunction

## Abstract

***Purpose:*** Manganism is a cognitive disorder take places in peoples are exposed to environmental manganese pollution. Overexposure to manganese ion (Mn^2+^) mainly influences central nervous system and causes symptoms that increase possibility of hippocampal damages.

***Methods:*** In this study rats were administrated by two different doses of MnCl2 and behavioral and physiological consequences were evaluated. We also investigated effects of E. Amoenum on Mn^2+^-imposed toxicity by behavioral, biochemical, immunoblotting and histological studies on hippocampus tissue.

***Results:*** Results showed metal overexposure increases oxidative stress mainly by lipid peroxidation and reactive oxygen species overproduction. Histological studies and caspase 3 analyses by immunoblotting revealed Mn^2+^ induced apoptosis from mitochondrial-dependent pathway in the presence of low metal dose. This study provides evidence that oral administration of E. amoenum extract inhibited manganese neurotoxicity by oxidative stress attenuation and apoptosis reduction that lead to improved depression like behavior. Plant extract also increased catecholamine content in Mn^2+^ treated hippocampus.

***Conclusion:*** As molecular and pathophysiological effects of E. amoenum, it could be considered as a pre-treatment for Parkinson and Parkinson like disorders in high-risk people.

## Introduction

Manganese (Mn) plays key role in mammalian brain development and function as a trace element.^1^ Mn^2+^ is cofactor of important enzymes such as glutamine synthetase, pyruvate decarboxylase, serine/threonine protein phosphatase I, Mn-superoxide dismutase and arginase, which are required for neurotransmitter synthesis, metabolism and antioxidant defense system.^2^ It is also the fourth most widely used heavy metal in the industry such as textile bleaching, leather tanning and iron, steel, potassium permanganate, hydroquinone, glass and ceramics production.^2,3^ By considering high application of Mn^2+^ in today’s life, exposing to this poisonous metal is predictable especially in miners and factory workers.^4^ Previous studies confirmed Mn^2+^ overdose causes Parkinson like disorder known as manganism that accompanied by tremors, odd movements, mask like face, and body stiffness that first observed in miners.^3^ The risk of Mn^2+^ exposure is not limited to miners or welders. The environmental accessibility and high Mn^2+^ concentration in water or food represent a source of contamination for the general population.^4^ As previous study, systemic injected Mn^2+^ mainly accumulated in central nervous system (CNS) and damaged it.^5^ Our prior results also showed acute dose of Mn^2+^ causes reduction of catecholamine level in the brain tissue by unknown mechanism.^6^ The main theories for damaging mechanisms of heavy metals are mitochondrial dysfunction and oxidative stress.^7^ Plants, especially *Echium amoenum*, are important source of antioxidants and cell protectants can be used for CNS toxicity.^8^ *E. amoenum* Fisch, a common traditional herbal medicine, is widely used as an effective treatment for tranquillizer, diaphoretic, cough, sore throat and pneumonia.^8,9^ Dried violet-blue petals of *E. amoenum* have been recently recognized as an important source of phenolic compounds like rosmarinic acid, cyaniding and delphinidin that could be extracted by water solvents.^10^ By considering molecular composition, it is believed that this plant has antibacterial, antioxidant, analgesic, anxiolytic, antidepressant and immunomodulatory properties.^10-13^ It has been shown that *E. amoenum* aqueous extract is effective treatment for obsessive-compulsive disorder and pancreatitis.^14,15^ Neuroprotective effects of cyanidin 3-glucoside, the most common anthocyanin in petals of this plant, have been investigated previously and results showed it can inhibit inflammation by blocking the c-Jun and NF-κB factors translocation into the nucleus.^16^ Therefor its possible *E. amoenum* aqueous extract prevents toxic effects induced by heavy metals such as Mn^2+^ in CNS.

The main goal of this study is evaluation of the MnCl_2_ toxicity in the rat hippocampus by biochemical analysis, behavioral assessment and histological studies. Hippocampus tissue plays an important role in hippocampal-dependent learning and memory, depressive-like behaviors and cognitive disorders similar to manganism, therefore we investigated Mn^2+^ toxicity in hippocampus tissue.^17,18^ Two different doses of metal were used to dose dependent assessment of physiological and biochemical parameters. This study also estimated *E. amoenum* aqueous extract improving effects on the neurotoxicity imposed by high dose of MnCl_2_.

## Materials and Methods

2,7 dichlorofluoresc indiacetate (DCFHDA), thiobarbituric acid, and *5*,*5*′-*Dithiobis* (2-nitrobenzoic acid) riboflavin and nitro blue tetrazolium were purchased from Sigma Chemical Company. All other solvents and chemicals were of the highest grade-commercially available.

### Experimental design and plant extraction

*In vivo* study was conducted on experimental animals and using adult male Wistar rats weighing 250-300 g obtained from the animal house of martyr portal. Animals with average age of 4.5-6 months were selected. Testing was carried out at temperature of 20-25 centigrade degree and that day duration was 12 hours and dark period was 12 hours. Municipal tap water was used as drinking water and animal feed as nutrition (compressed feed). We have 4 experimental groups in this study and 8 rats in each group. The first group was daily injected by physiological saline (0.9 % NaCl) for 15 days, the second group was daily injected by 10 mg/kg MnCl_2_ in saline as vehicle during 15 days, third group was injected daily by 15 mg/kg MnCl_2_ in saline for 15 days and fourth group was administrated by 15 mg/kg MnCl_2_ + 5 mg/kg *E. amoenum* extract for same time duration. MnCl_2_ and extract doses determined according to previous study^19^ and some experiments (data not shown). Although there are many methods for plant extract preparing, but few scientific reports are available in the literature on lyophilized extracts of fresh violet petals of *E. amoenum*, as this type of extraction causes better antioxidant and cell protective potential in rats.^20^ Sample was collected after approval of agricultural experts. The fresh violet petals of *E. amoenum* were thoroughly washed with tap water and its juice was obtained using a blender. After obtaining the juice, it was lyophilized to get the dry powder using freeze dryer. We used 5 mg/kg of plant extract in saline for orally administration of rats before 15 mg/kg MnCl_2_ intraperitoneally injection.

### Behavioral assessment 

#### Forced swimming test (FST)

As previous standard protocol,^21^ rats were placed in a transparent plexiglass cylinder (20 cm diameter and 50 cm height) filled with warm water (25 °C and 30 cm depth). The classical procedure uses a two-day protocol. The first day of habituation, the rats were forced to swim for 15 min; 24 h after, on the test day three categories of behavioral activity (climbing, swimming and immobility) were recorded during the 5 min test period. Immobilization time considered as time between introduction of a rat into the pool and making only those movements necessary to keep its head above water without struggling. Immediately after each experience rats were dried and kept warm before returning to their home cage.

#### Sucrose preference test (SPT)

SPT estimates hedonia (pleasure-seeking) or its deficient (anhedonia) by monitoring preference of rats to sucrose water.^22^ Rats were placed in individual cages with food and water. At first, rats were adapted to having two water bottles in the cage lid for 72 hours and their position was randomly changed as many times as possible to avoid a place preference. The bottles were fitted with ball-bearing sipper tubes to prevent fluid leak. After this acclimation, rats had the free choice of either bottle for water drinking. Then one of the bottles filled with 1% sucrose solution during 48 hours test. Water and sucrose solution intake was measured daily. The locations of two bottles were switched daily to reduce side bias. Sucrose preference was calculated as follows: 100 * [sucrose consumption (g)/(sucrose consumption (g) + water intake (g))] and averaged over the 2 days of testing.

#### Reactive oxygen species (ROS) measurement

ROS generation was measured according to the methods of Keston and Brandt^23^ and Lebel et al.^24^ with some modifications. The method used to measure the oxidative conversion of DCFH-DA to dichlorofluorescin (DCFH) as a fluorescent compound. Hippocampus homogenates were diluted 1:10 in buffer to obtain a concentration of 5 mg tissue/ml. Then the homogenates were pipetted into 24-well plates (0.45 ml/ well) and allowed to warm to room temperature for 5 min. At that time, 5 µl of DCFH-DA (10 µM final concentration) was added to each well and the plates preincubated for 15 min at room temperature to allow the DCFH-DA to be incorporated into any membrane-bound vesicles and the diacetate group cleaved by esterases. After the preincubation, 50 µl of the appropriate concentration of Fe^2+^ was added to the wells. After 30 min, DCFH-DA converted into non-fluorescent DCFH, which reacts with ROS to form the fluorescent product DCF. DCF fluorescence was determined at 485 nm excitation and 530 nm emission using a (Perkin Elmer luminescence spectrometer LS 55) fluorescence spectrophotometer. The slit width was 5 nm for both excitation and emission. Background fluorescence (conversion of DCFH to DCF in the absence of homogenate) was corrected by the inclusion of parallel blanks. ROS content was expressed as DCF fluorescence/mg protein/min in comparison with control. Protein concentration in homogenates was determined using Bradford method^25^ and did not differ between groups.

#### Lipid peroxidation (LPO) measurement

Hippocampus tissue samples were used for measurement of lipid peroxidation as a marker of oxidative stress.^26^ Tissue was separated after anesthesia and homogenized with ice cold buffer containing 0.15 M KCl to obtain 1:10 (w/v) homogenates. Aliquots of homogenate (1 ml) were incubated at 37°C for 3 h in a shaker. Then, 1ml of 10% aqueous trichloroacetic acid (TCA) was added and mixed. The mixture was then centrifuged at 800 g for 10 min. Then, supernatant (1 ml) was mixed with 1ml of 0.67% thiobarbituric acid and placed in a boiling water bath for 10 min. The mixture was cooled and diluted with 1ml distilled water. The absorbance of the solution was then read using spectrophotometer at 532 nm. In this process malondialdehyde (MDA) as a final product of lipid peroxidation reacts with tiobarbitouric acid (TBA) (this reaction completes in 100°C) and releases TBARS that absorbs 532 nm light.^27^ Results were expressed as percent of MDA production compared to the control.

#### Antioxidant enzymes assay

#### Superoxide dismutase (SOD)

SOD activity was measured spectrophotometrically according to the riboflavin/nitro blue tetrazolium (NBT/RF) assay method.^28^ This indirect method involves the inhibition of NBT reduction. In the NBT/RF method, SOD competes with NBT for O_2_- generated by the RF under illumination. The 1.5 ml reaction mixture (50 mM KH_2_PO_4_, pH 7.8, 0.1 mM EDTA), 2 mM riboflavin and 57 μM NBT) were used. Since generation of O_2_- radicals in the NBT/RF assay is driven by light, samples were subsequently illuminated from above for 15 min by 4 fluorescence tubes (40 W, 30 cm distance) giving 199 μmol photons m^2^ s^−1^. Afterwards, absorbance was measured at 560 nm. Fifty percent inhibition was calculated by regression using the linear part of a natural semi-log curve after which the specific activity was calculated. One unit of SOD activity was defined as the amount of enzyme that inhibits 50% of NBT photochemical reduction.

#### Catalase (CAT)

Catalase activity was measured following the method of Aebi with minor modifications.^29^ The principle of this method was based on the hydrolyzation of H_2_O_2_ and decreased absorbance at 240 nm. The conversion of H_2_O_2_ into water and 1/2 oxygen per minute at 25 °C and phosphate buffer (pH 7) was considered to be the enzyme reaction velocity.

#### Catecholamine measurement 

24 hours after last treatment rats were anesthetized with ketamine/xylazine and hippocampus was separated from sculpture. Fresh tissue or tissue that had been frozen in liquid nitrogen and stored at −80 °C were homogenized in cold 0.05 N HClO_4_ containing dihydroxybenzylamine as internal standard. The supernatant after 15 min centrifugation at 12000 g was processed according to Felice et al., except that 0.1 N HClO_4_ was used to elute the amines from the alumina.^30^ Catecholamine converts to fluorescent substance in alkaline environment and in the presence of ascorbic acid and iodine as a strong oxidant.^30^ Fluorescence studies were carried out on a Perkin Elmer luminescence spectrometer LS 55. The excitation wavelength was set at 405 nm and the emission spectra were recorded in 515 nm. Excitation and emission slit were both set at 5 nm.

#### Western blotting analysis

Caspase 3 and caspase 9 have important role in mitochondria dependent and independent apoptosis pathway respectively so were selected to be quantified via western blot by using specific antibodies (rat specific anti-Caspase-3 and anti-Caspase-9 antibodies purchased from Abcam company). Western blotting was carried out according to our previous study,^31^ after SDS-PAGE, the proteins were transferred onto PVDF membrane actively at 140 V for 1.5-2 h in the transfer buffer. After completion of the transfer and blocking, membrane was probed with the primary and secondary specific antibodies and was washed four times in TBST (50 mM Tris, pH 7.5, 150 mM NaCl, 0.05 % Tween 20) between incubations. Bands containing specific proteins were visualized using an ECL detection system according to the manual. Anti β-actin (1:1,000) (Cell Signaling Technology) was used as a housekeeping control. The density was calculated through ImageJ 1.46r; Java 1.6.0_20 software for each band.

#### Statistical evaluation

All values were expressed as the mean ± standard error of mean (S.E.M). Data was analyzed using one-way ANOVA followed by the post-hoc Duncan multiple range test for analysis of biochemical data using SPSS version 11. Differences were considered significant at p < 0.05.

## Results and Discussion

Extensive application of the Mn^2+^in industry and its pollution in the environment, expose human and animal to the manganese neurotoxicity.^4^ This study examines toxic effects of MnCl_2_ on Wistar rats as a suitable model. Therefore we administrated rats by 10 and 15 mg/kg MnCl_2_ intraperitoneally and assessed behavioral parameters of depression such as body weight dynamic, sucrose preference and immobilizing time in forced swimming test. We also measured ROS, LPO and oxidative stress barriers such as catalase and SOD for examine the role of Mn^2+^ in oxidative damages in rat brain. Catecholamine as an important neurotransmitter in health and normal application of brain was measured. MnCl_2_ role in cell death was estimated by measuring of the apoptosis involved proteins and tissue sections analysis. We also assessed *Echium amoenum* extract effects on Mn^2+^-induced neurotoxicity as an important traditional medicine grows in northern part of Iran.^9,10^ This plant has been recognized as an important source of phenolic compounds like rosmarinic acid, cyanidin and delphinidin which potentially have chemoprotective effects against toxic metals that are increasing in life today.^4,13^ By considering chemical nature of antioxidants and biocompatibility, saline was used in extraction process. Manganese toxicity upon overexposure (manganism) was reported to be accompanied by Parkinson and depression like behavior assigns in miners and welders.^2,4^ By considering cognitive disorder as main problem in patients suffer from manganism, its predictable hippocampus is one of the affected tissues in CNS that has not been studied previously.^17,18^

### Behavioral assessment 

#### Body weight

**Figure 1:**
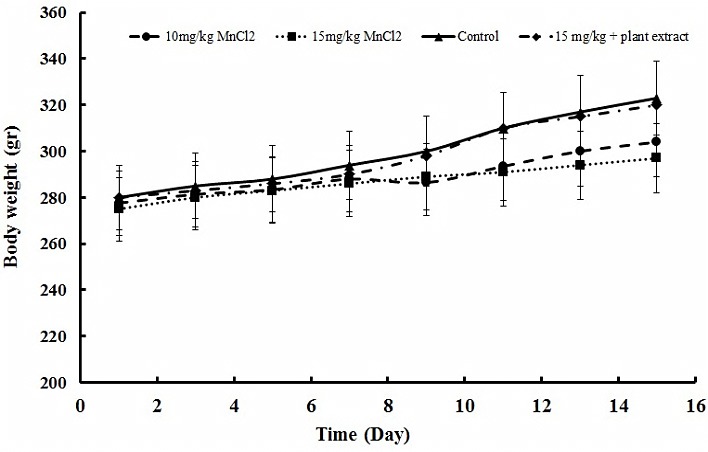
Body weight dynamic during the experimental period. Results showed weight loss in manganese treated rats that improved by oral administration of *E. amoenum* extract. Each data indicates the mean ± S.E.M.

Body weight dynamics is a sensitive indicator for chemical toxicants.^32^ Previous studies have shown that administration of toxic nanoparticles such as AgNPs and ZnNPs significantly decreased body weight growth rate in rats.^32,33^ Therefore to investigate whether exposure to MnCl_2_ could be considered as a global health issue, we monitored the mortality rate, food consumption, water intake, and body weight dynamic of experimental groups during study. Results showed water/food and survival of rats were not changed significantly in treated rats during experiment. But body weight progressive curve was affected by both doses of MnCl_2_ and metal treated animals showed a slow increase in body weight. The rats were injected by MnCl_2_ revealed growing body weight with a mean ± S.E.M. of 304.7 ± 15.2 g in 10 mg/kg Mn^2+^-treated rats, 297.2 ± 10.4 in 15 mg/kg Mn^2+^-treated rats and 323.4 ± 12.9 g in control after 15 days. As Figure 1, slop of the body growing curve is dose dependent and has an inverse relationship with Mn^2+^ dose. Interestingly *E. amoenum* extract improved Mn^2+^ effect, as Figure 1 showed. Body weight of rats that received plant extract in addition to high dose of Mn^2+^, increases near to the control, 320 ± 13.1 g at the end of experiment.

#### Forced swimming test (FST)

**Figure 2:**
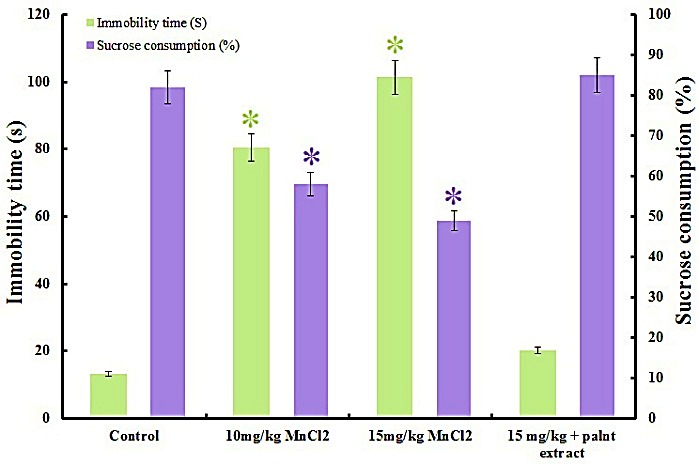
Depression like behavior test. Increased immobilization time and decreased sucrose preference confirmed depression like behavior in rats that received manganese dose dependently. Results showed *E. amoenum* extract compensates metal toxic effects. Each value indicates the mean ± S.E.M. Asterisk symbols showed significant changes by P<0.05.

FST was done to assess depressive-like behavior^21^ as a result of MnCl_2_ injection. Recorded immobility time for 10 mg/kg MnCl_2_ treated rats is 80.54 ± 4.65 sec, for 15 mg/kg is 101.34 ± 6.43 sec and in control is 15.20 ± 3.23 sec. Therefore Mn^2+^ treatment significantly increased immobility time compared to the control that refer to depression in Mn^2+^treated rats dose dependently. Figure 2 revealed daily treatment of rats that received 15 mg/kg Mn^2+^ with plant extract reduced immobilization time significantly from 80.54 ± 4.65 sec to 20.22 ± 3.43 sec that approved aqueous extract of *E. amoenum* relieved depression like behaviors signs. Plant extract slightly increased immobility time in control rats but it’s not significant (P<0.05).

#### Sucrose preference test (SPT)

Sucrose preference test is one of the most commonly used assays for depression in rodents,^22^ so we used this test to anhedonia evaluation in Mn^2+^ or/and plant extract treated rats and control. Results showed sucrose consumption decreased significantly in Mn^2+^ received rats dose dependently (Figure 2). Sucrose intake in rats received 10 mg/kg is 58.02 ± 5.13 %, in rats administrated with 15 mg/kg is 49.32 ± 4.29 % and in control rats is 82.16 ± 8.56 %. Decreased sucrose preference refers to depression like behavior in rats as a result of Mn^2+^injection especially in rats that received high dose. Figure 2 also showed decreased sucrose consumption was improved by plant extract in rats that received high dose of metal (85.45 ± 4.76 %). Control rats which received an equal dose of plant extract don’t show significant differences in sucrose intake (data not shown).

### Oxidative stress in hippocampus tissue 

Noticed depression like behavior and previous studies approved one of the important targets for metals toxicity is CNS.^5-7^ To investigate whether the administration of toxic doses of Mn^2+^ creates oxidative stress in hippocampus tissue, we studied ROS content and oxidative barrier enzymes activity in four experimental groups.

#### ROS measurement

**Figure 3:**
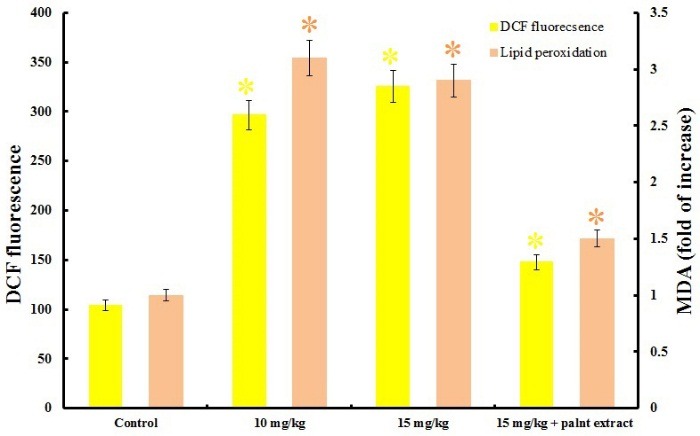
Oxidative stress investigation. Rising of the DCF fluorescence in hippocampus related to metal treated rats refer to ROS overproduction. Increased ROS molecules causes lipid peroxidation that showed by high malondialdehyde content. Interestingly increased oxidative stress attenuated by *E. amoenum* extract. Each data indicates the mean ± S.E.M. Asterisk symbols showed significant changes by P<0.05.

ROS are chemically reactive molecules mainly contain super oxide, peroxide and hydroxyl groups that basically are produced by mitochondria during normal metabolism in low concentration but acute stress increases it by causing mitochondrial dysfunction.^34^ Therefore, ROS measurement will give a useful report from oxidative state of the hippocampus tissue‏ related to experimental groups. Figure 3 revealed Mn^2+^ induced high DCF fluorescence intensity that refers to ROS overproduction in hippocampus tissue. 10 mg/kg MnCl_2_ administration raised ROS level more than 2.5 folds and by increasing metal dose to 15 mg/kg, ROS elevated to more than 3 folds rather than control. Therefore MnCl_2_ administration causes harsh oxidative stress in hippocampus tissue by overproduction of ROS molecules (Figure 3). Previous studies showed heavy metals damaged mitochondria and interrupted respiratory chain that lead to reactive molecules production.^35^ ROS content of MnCl_2_ treated hippocampus decreased significantly by plant extract. The antioxidant capacity of phenolic compounds in plant extract is attributed to their ability in metal ions and ROS molecules chelating, so protect cell compounds from oxidation.^31^ Apoptosis and necrosis in living system are main outcomes of ROS overproduction.^36^ Plant effects on ROS level of control rats are not significant that approved *E. amoenum* possibly doesn’t affect healthy function of mitochondrial.

#### Lipid peroxidation (LPO) evaluation 

LPO is the most popular indicator of oxidative stress can be used as a marker of cell membrane injuries also.^26,37^ Overproduced reactive molecules attack to the cell membrane lipids and damage it by lipid peroxidation.^38^ Malondialdehyde (MDA) is the most known secondary products of lipid peroxidation could be evaluated by thiobarbituric acid method.^27^ The rats which received both doses of metal showed increased MDA about 3 folds than control, that were initially caused by increased free radicals (Figure 3). As Figure 3, plant extract treatment reduces MDA level (about 2 folds) in hippocampus tissue of rats that received 15 mg/kg MnCl_2_. Therefore MDA level increased in the presence of MnCl_2_ dose dependently that refers to elevated lipid peroxidation as a result of metal toxicity (Figure 3). Plant extract contains antioxidant molecules that diminished ROS level and consequently reduced MDA in metal treated rats.

#### SOD and catalase activity measurement 

**Figure 4:**
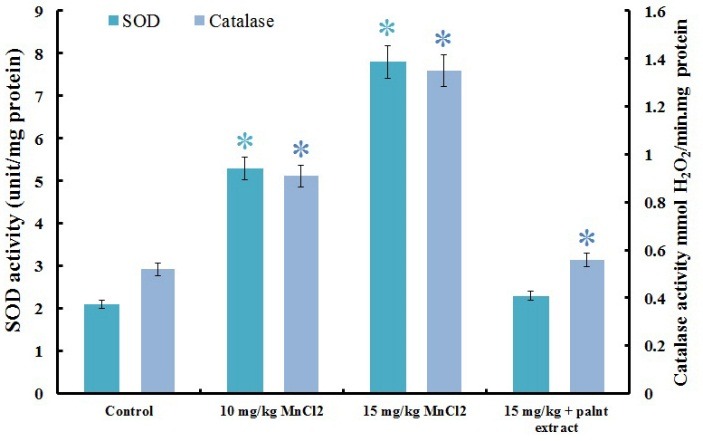
Antioxidant enzyme assessment. Oxidative stress imposed by manganese toxicity causes improving of the superoxide dismutase and catalase enzymes activity in hippocampus tissue as an adaptation to toxic stress. Antioxidant enzymes activity decreased in rats that received plant extract. Each value indicates the mean ± S.E.M. Asterisk symbols showed significant changes by P<0.05.

Hippocampus tissue samples were used for measurement of superoxide dismutase (SOD) and catalase (CAT) activities as the most popular antioxidants barrier in biological systems. Since the Mn^2+^ treated rats produce an oxidative stress which could be exhausted by the antioxidative ability of SOD and CAT.^39^ Therefore, we estimated both enzyme activities in four experimental groups. Activities of both enzymes were increased in rats who received 10 and 15 mg/kg MnCl_2_ (Figure 4). SOD and CAT activity that induced by Mn^2+^, were reduced by plant extract near to the control. As Figure 4, 15 mg/kg MnCl_2_ causes activation of SOD to 7.8 unit and catalase to 1.35 mmol/min but plant extract decreased it to 2.3 unit and 0.56 mmol/min respectively, while activity of enzymes in control rats are 2.1 unit and 0.52 mmol/min for SOD and catalase respectively. Difference between control group, rats that received 15 mg/kg MnCl_2_ + plant extract and normal rats that received plant extract are not significant (P<0.05). By considering ROS overproduction in Mn^2+^ administrated rats, increased activity of the SOD and catalase (Figure 3) refers to an adaptation of hippocampus cells to neutralize the extra produced oxidant compounds.^40^ The increased SOD activity possibly resulted from SOD overexpression that is regulated by Mn^2+^ as essential cofactor of this enzyme.^2^

### Catecholamine content of hippocampus tissue

Catecholamines, including dopamine and norepinephrine, are most the important neurotransmitters that mediate a variety of functions in CNS, such as motor control, cognition, emotion, memory processing, and endocrine modulation.^41^ Dysfunctions in catecholamine neurotransmission are related to some neuropsychiatric disorders specially Parkinson disease and epilepsy.^31^ Similar neuropsychiatric signs in Parkinson disease and manganism possibly are caused by equal molecular events.^2^ Therefore catecholamine content of hippocampus tissue was compared between experimental groups as follow: 10 mg/kg MnCl_2_, 142.43 ± 12.52 ng/mg protein; 15 mg/kg MnCl_2_, 91.45 ± 4.52 ng/mg protein; 15 mg/kg MnCl_2_ + plant extract, 250.45 ± 12.34 ng/mg protein and control rats, 210.32 ± 10.23 ng/mg protein. Decreased catecholamine may be caused by increased dopaminergic cell death in the presence of metal ions.^7^ Diminished catecholamine was returned near to (even more than) the control by plant extraction treatment, while these kinds of neurotransmitters have dual action (Neurotoxic and neuroprotective) and according to previous experiments, high doses of catecholamine induces apoptosis in the neurons.^42^ Control rats that received plant extract showed increase in catecholamine content (224.41 ± 14.29 ng/mg protein) but it’s not significant and does not accompanied with abnormal neurobehaviours. Relieving effects of *E. amoenum* in molecular level especially catecholamine rising, finally lead to improved depression like behavior in rats treated by toxic doses of metal as discussed above.

### Caspase 9 and caspase 3 analysis

**Figure 5:**
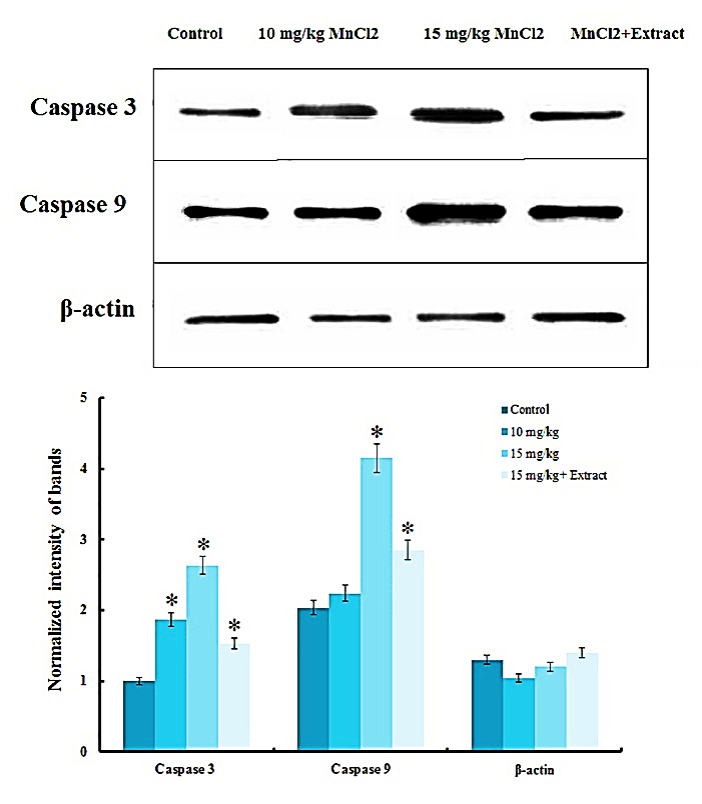
Immunoblotting studies. Up-regulation of caspase 3 and 9 during manganese intoxication refers to increased apoptosis in metal received rats. *E. amoenum* extraction significantly decreased neurodegeneration in hippocampus tissue. The intensity of bands was quantified by ImageJ software. The data were expressed as mean ± S.E.M of three independent experiments. Asterisk (*) was used to denote statistical significance (P<0.05).

Raised oxidative stress and reduced catecholamine possibly cause cell death in metal treated hippocampus. Catecholamine level of brain is important in healthy function and survival of neurons and decreased catecholamine lead to neurodegeneration in some neurological disease.^42^ ROS overproduction was caused by mitochondrial dysfunction or/and inefficient antioxidant barrier that lead to mitochondrial-dependent and –independent apoptosis with different molecular mechanisms.^43^ Caspase 9 involves in mitochondrial-independent and caspase 3 participates in mitochondrial-dependent apoptosis.^44,45^ Our experiments revealed in rats that received 10 mg/kg MnCl_2_ only caspase 9 increased significantly but in rats treated by 15 mg/kg MnCl_2_ both of the caspase 3 and caspase 9 increased in hippocampus (Figure 5). These results confirmed more sensitivity of the mitochondria against metal toxicity. Manganese overexposing well documented to result in a disrupted Fe^2+^homeostasis that lead to mitochondrial dysfunction.^44^ As Figure 5, increased expression of the caspase 3 and 9 that induced by 15 mg/kg of metal was improved by oral administration of plant extract.

### Histological studies 

**Figure 6:**
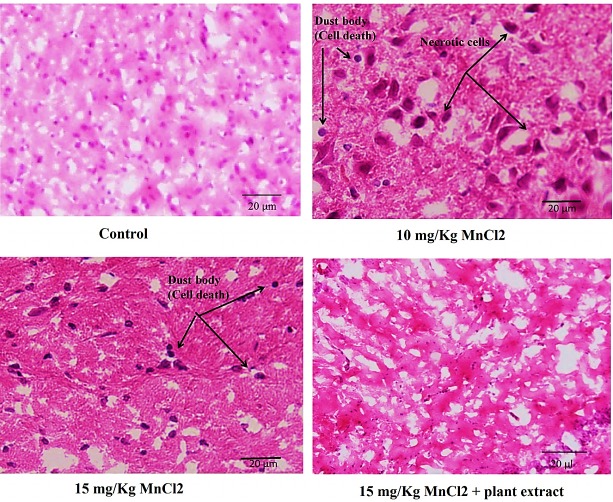
Histological studies. Hematoxylin/eosin staining of hippocampus sections revealed presence of the apoptotic and necrotic cells in MnCl_2_ treated rat hippocampus rather than control. Result showed oral administration of *E. amoenum* extraction significantly decreased apoptotic and necrotic cells in hippocampus.

The biological significance and toxicological importance of any changes which are found between tissue section in control and experimental groups have been considered as biochemical results confirmation. Therefore after the end of experimental time course, rats were anesthetized and brain tissue separated from scalp. Tissue samples were treated by formalin for fixation and stained by hematoxilin-eosin method and then studied by light microscope.^46^ Results showed presence of necrotic and apoptotic cells in tissues were administrated by 10 and 15 mg/kg MnCl_2_ (Figure 6). Early apoptotic nuclei have a condensed appearance that frequently seen in MnCl_2_ administrated tissue especially in 15 mg/kg MnCl_2_ received rats. Increased apoptosis in the metal treated rats accompanied by decreased level of the catecholamine may be due to catecholamine positive role in cell survival or catecholamine producing cell death in Mn^2+^neurotoxicity. Histology results also were confirmed by elevated level of caspase 3 and 9 in intoxicated rats. As Figure 6, amounts of condensed apoptotic and deformed necrotic cells reduced in tissues related to rats received plant extract+15 mg/kg MnCl_2_ that accompanied by decreased caspases and improved behavioral abnormalities also.

## Conclusion

Pathophysiological signs of manganism in human and animal models suggest hippocampus as a possible affected tissue. Our biochemical results approved hash oxidative damages in the presence of MnCl_2_ doses that attenuated by plant extract. Increased expression of the caspase 9 in low dose of Mn^2+^ revealed, metal toxicity causes mitochondrial dysfunction at first and then induces oxidative damages that lead to mitochondrial independent apoptosis in the presence of high metal dose (caspase 3 upregulation). Despite the prevalent use of *E. amoenum* as an antidepressant, there are no pharmacological data to support such effects. Our molecular and biochemical studies confirmed *E. amoenum* extract inhibited apoptosis from both described pathways possibly by ROS molecules scavenging, mitochondrial dysfunction improving and metal ions trapping. All of the identified beneficial effects or possibly uncharacterized mechanisms lead to decreased depressive behaviors. Investigated therapeutic effects of *E. amoenum* on Mn^2+^ neurotoxicity revealed this plant could be considered in antidepressant drug design and as supplement against all of the metals toxicity or oxidative damages. By considering physiological and molecular similarities between manganism and Parkinson disease and also *E. amoenum* role in catecholamine overproduction, this plant could be used as natural co-treatment in associated diseases.

## Ethical Issues

All the experimental works were approved by the Ethical Committee of Isfahan University of Medical Science (Isfahan, Iran) and conform to the European Communities Council Directive of 24 November 1986 (86/609/EEC).

## Conflict of Interest

All of the Authors have no conflict of interest to declare.
